# Identification of a novel m5C/m6A-related gene signature for predicting prognosis and immunotherapy efficacy in lung adenocarcinoma

**DOI:** 10.3389/fgene.2022.990623

**Published:** 2022-09-30

**Authors:** Yiming Ma, Jun Yang, Tiantai Ji, Fengyun Wen

**Affiliations:** ^1^ Department of Medical Oncology, Cancer Hospital of China Medical University, Liaoning Cancer Hospital and Institute, Shenyang, China; ^2^ Department of Gastrointestinal Surgery, The First Hospital of China Medical University, Shenyang, China; ^3^ Department of Radiotherapy, Cancer Hospital of China Medical University, Liaoning Cancer Hospital and Institute, Shenyang, China

**Keywords:** LUAD, immunotherapy, drug sensitivity, RNA methylation, prognosis

## Abstract

Lung adenocarcinoma (LUAD) is the most prevalent subtype of non-small cell lung cancer (NSCLC) and is associated with high mortality rates. However, effective methods to guide clinical therapeutic strategies for LUAD are still lacking. The goals of this study were to analyze the relationship between an m5C/m6A-related signature and LUAD and construct a novel model for evaluating prognosis and predicting drug resistance and immunotherapy efficacy. We obtained data from LUAD patients from The Cancer Genome Atlas (TCGA) and Gene Expression Omnibus (GEO) datasets. Based on the differentially expressed m5C/m6A-related genes, we identified distinct m5C/m6A-related modification subtypes in LUAD by unsupervised clustering and compared the differences in functions and pathways between different clusters. In addition, a risk model was constructed using multivariate Cox regression analysis based on prognostic m5C/m6A-related genes to predict prognosis and immunotherapy response. We showed the landscape of 36 m5C/m6A regulators in TCGA-LUAD samples and identified 29 differentially expressed m5C/m6A regulators between the normal and LUAD groups. Two m5C/m6A-related subtypes were identified in 29 genes. Compared to cluster 2, cluster 1 had lower m5C/m6A regulator expression, higher OS (overall survival), higher immune activity, and an abundance of infiltrating immune cells. Four m5C/m6A-related gene signatures consisting of HNRNPA2B1, IGF2BP2, NSUN4, and ALYREF were used to construct a prognostic risk model, and the high-risk group had a worse prognosis, higher immune checkpoint expression, and tumor mutational burden (TMB). In patients treated with immunotherapy, samples with high-risk scores had higher expression of immune checkpoint genes and better immunotherapeutic efficacy than those with low-risk scores. We concluded that the m5C/m6A regulator-related risk model could serve as an effective prognostic biomarker and predict the therapeutic sensitivity of chemotherapy and immunotherapy.

## Introduction

Lung cancer is the most prevalent cause of cancer-related death worldwide, with non-small cell lung cancer (NSCLC) accounting for approximately 85% of all cases (Jin et al., 2019). As the most common subtype of NSCLC, lung adenocarcinoma (LUAD) has a 5-year survival rate of 15%–20% because of the migration and invasion of cancer cells (Kim et al., 2019; Cai et al., 2020). Although targeted therapy and immunotherapy have made progress (Santarpia et al., 2020), some LUAD patients still have poor therapeutic effects, which lead to relapse and progression of cancer. Therefore, it is essential to develop an appraisal procedure to evaluate the prognosis and guide personalized treatment strategies for LUAD.

Immunotherapeutic treatment is becoming a novel strategy for modern tumor treatment. As a promising immunotherapy modality, immune checkpoint inhibitors (ICI) such as PD1 blockade have shown clinical benefits for LUAD and other cancer types (Steven et al., 2016; Suresh et al., 2018; Li et al., 2019a). Anti-PD1 and anti-PDL1 agents are expected to become effective treatment options for advanced-stage LUAD. Some indicators, such as genomic demethylation, microsatellite instability, mismatch repair deficiency, and tumor mutational burden (TMB), have been demonstrated to have a predictive potential for patients with cancer (Benayed et al., 2019; Jung et al., 2019; Fujimoto et al., 2020). However, a reliable biomarker for predicting immunotherapy response is still lacking in clinical practice.

Since RNA modification was first discovered, more than 100 different RNA modifications have been identified in eukaryotes, including N6-methyladenosine (m6A), 5-methylcytidine (m5C), and N1-methyladenosine (m1A) (Lorenz et al., 2020). M6A and m5C were the most prevalent mRNA modification patterns (Thomas et al., 2019). M6A is a universal methylated modification pattern that exerts gene expression by regulatory proteins acting as writers, erasers, and readers (Dai et al., 2021). Recent studies have shown that m6A is involved in the progression of obesity (Wang et al., 2020), periodontitis (Zhang et al., 2021), and renal fibrogenesis (Liu et al., 2020a). With its high abundance in eukaryotes, m5C also plays a crucial role in regulating gene expression (Yang et al., 2020). Previous studies have revealed that RNA modification regulators are closely related to the progression of human cancers, such as lung, breast, and brain cancers (Deng et al., 2018; Li et al., 2019b). Moreover, a study showed that four types of RNA modification writers might play a vital role in the tumor microenvironment (TME), targeted therapy, and immunotherapy in colorectal cancer (Chen et al., 2021a). However, the functional importance of the m5C/m6A-related genes in LUAD remains unclear.

**FIGURE 1:**
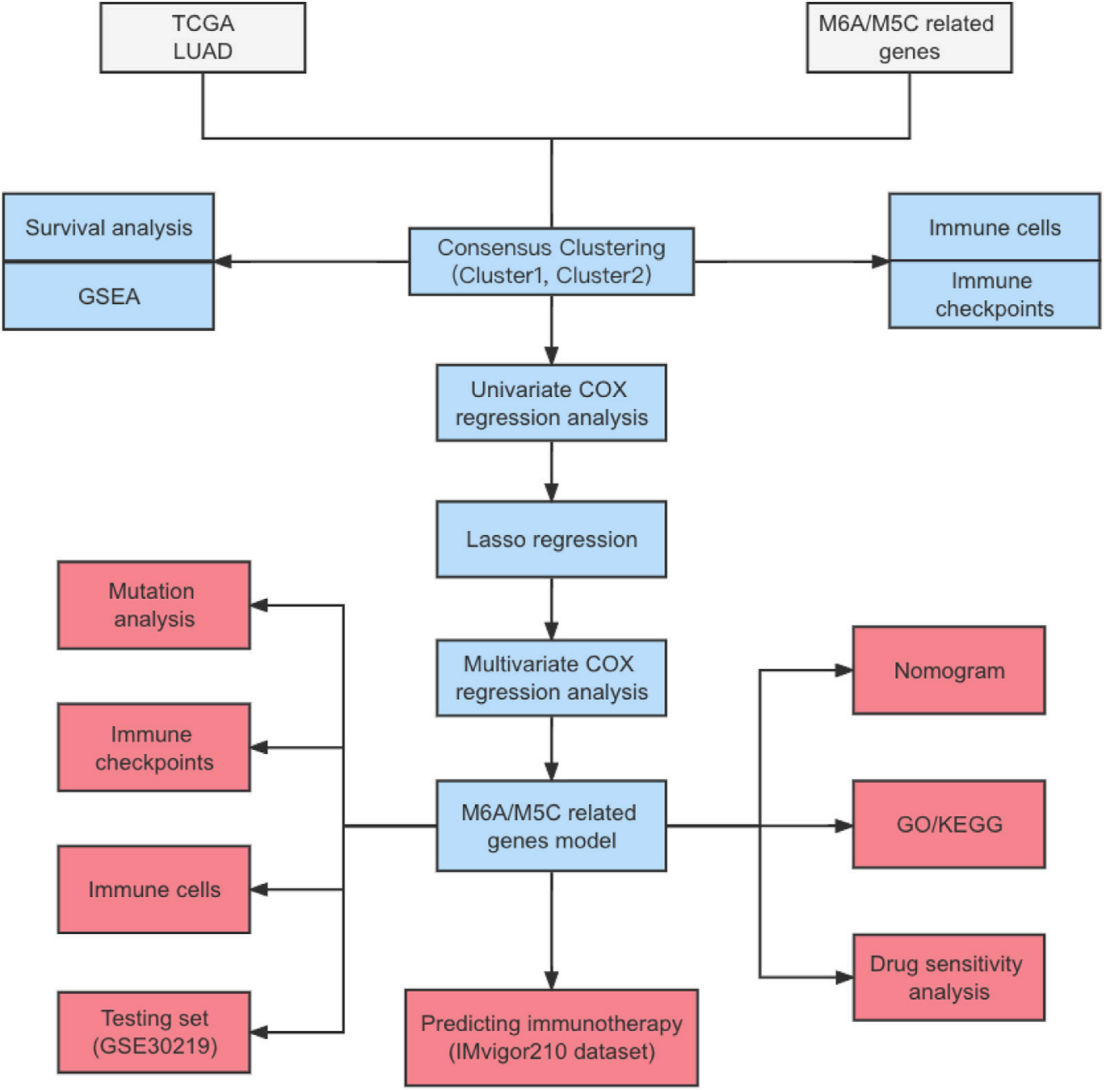
Flowchart.

With the development of genome sequencing and screening techniques, gene expression profiles have been used to identify prognostic genes as novel biomarkers for different types of cancer. In this study, we showed the expression levels, correlations, and mutation profiles of m5C/m6A-related genes and identified distinct m5C/m6A-related subtypes in The Cancer Genome Atlas (TCGA)-LUAD cohort. Using univariate Cox regression, lasso regression, and multivariate Cox regression, four prognostic factors were screened out, and risk models were constructed using these genes and validated using GSE30219. The predictive immunotherapy value of the risk score was evaluated using the IMvigor210 dataset (the whole process of data analysis was described in Figure 1). In conclusion, the m5C/m6A-related risk score was a significant prognostic factor in LUAD and a potential biomarker for stratifying LUAD patients benefiting from immunotherapy.

## Materials and methods

### Data collection and preprocessing

We collected publicly available gene expression data for LUAD cohorts and corresponding clinical information from TCGA and Gene Expression Omnibus (GEO) databases. Patients with LUAD with complete survival information were included in further analyses (Supplementary Table S1). The RNA sequencing data on 497 TCGA-LUAD samples were downloaded using the R package TCGAbiolinks (Colaprico et al., 2016) as the training set. Masked somatic mutation and copy number variation (CNV) data on TCGA-LUAD samples were downloaded from TCGA. The expression profiles of 85 cases from GSE30219 were downloaded as the test set. Immune checkpoint inhibitor treatment in patients with available expression data was also used in our research study. The IMvigor210 dataset, in which advanced urothelial cancer patients were treated with anti-PD-L1 agents, was also applied for analyses evaluating immunotherapy response. RNA-seq data and clinical details from the IMvigor210 dataset were acquired using the R package IMvigor210CoreBiologies (Mariathasan et al., 2018).

### Unsupervised consensus clustering for m5C/m6A subtypes

We collected m5C/m6A-related genes from previous studies on RNA modifications (Zhang et al., 2021; He et al., 2022). These 23 m6A-related and 13 m5C-related genes are listed in Table 1. We then analyzed the differences in expression between normal and LUAD samples using the Wilcoxon rank-sum test. The protein–protein interaction (PPI) network was constructed using m5C/m6A-related genes in the STRING database and visualized by Cytoscape software (Mering et al., 2003; Shannon et al., 2003). Consensus clustering was utilized to identify m5C/m6A subtypes with differentially expressed m5C/m6A regulators. The process was completed with a clustering algorithm in the ConsensusClusterPlus R package (Wilkerson and Hayes, 2010). We used t-distributed stochastic neighbor embedding (t-SNE) to show the distribution of different m5C/m6A subtypes using the R package Rtsne (Linderman and Steinerberger, 2019).

**TABLE 1:** Genes related with m6A and m5C.

Gene	RNA methylation	Type
*METTL3*	m6A	Writer
*METTL14*	m6A	Writer
*WTAP*	m6A	Writer
*VIRMA*	m6A	Writer
*RBM15*	m6A	Writer
*RBM15B*	m6A	Writer
*CBLL1*	m6A	Writer
*ZC3H13*	m6A	Writer
*FTO*	m6A	Eraser
*ALKBH5*	m6A	Eraser
*YTHDF1*	m6A	Reader
*YTHDF2*	m6A	Reader
*YTHDF3*	m6A	Reader
*YTHDC1*	m6A	Reader
*YTHDC2*	m6A	Reader
*HNRNPC*	m6A	Reader
*HNRNPA2B1*	m6A	Reader
*IGF2BP1*	m6A	Reader
*IGF2BP2*	m6A	Reader
*IGF2BP3*	m6A	Reader
*FMR1*	m6A	Reader
*ELAVL1*	m6A	Reader
*LRPPRC*	m6A	Reader
*TRDMT1*	m5C	Writer
*NSUN2*	m5C	Writer
*NSUN3*	m5C	Writer
*NSUN4*	m5C	Writer
*NSUN5*	m5C	Writer
*NSUN6*	m5C	Writer
*NSUN7*	m5C	Writer
*DNMT1*	m5C	Writer
*DNMT3A*	m5C	Writer
*DNMT3B*	m5C	Writer
*YBX1*	m5C	Eraser
*TET2*	m5C	Eraser
*ALYREF*	m5C	Reader

### Evaluation of infiltrating immune cells, immune functions, and immune checkpoints

Based on the markers of the 28 types of immune cells (Supplementary Table S2), we used single-sample gene set enrichment analysis (ssGSEA) to calculate immune cell scores using the R package GSVA (Hänzelmann et al., 2013). We also analyzed the LUAD gene expression matrix using the R package CIBERSORT and obtained the abundance of 22 types of immunocytes (Newman et al., 2015). We downloaded an immunologically relevant gene list (Supplementary Table S3) from the ImmPort database (https://www.immport.org/resources). The immune function scores of LUAD samples were calculated using the ssGSEA algorithm. The differences in the expression of 30 immune checkpoint genes in the different groups were also analyzed in our study (Wang et al., 2021a).

### Construction and validation of the m5C/m6a-related prognostic risk model

The Wilcoxon rank-sum test was used to identify differentially expressed m5C/m6a-related genes between the normal and LUAD groups. To distinguish the m5C/m6a-related genes related to LUAD prognosis, univariate Cox regression was used to analyze the relationships between the differentially expressed genes and overall survival and filtering genes with *a p*-value < 0.1. Least absolute shrinkage and selection operator (LASSO) was performed to compress the number of genes and remove collinearity using the R package glmnet (Simon et al., 2011). Finally, we screened out prognostic m5C/m6a-related genes by multivariate Cox regression analysis and constructed a prognostic model. The risk score was calculated as follows: risk score = (coef-Gene1 × exp-Gene1)+(coef-Gene2 × exp-Gene2)+…+(coef-Gene × exp-Gene) (exp: gene expression level, coef: coefficients). We divided LUAD samples into low-risk and high-risk groups based on the median risk score and analyzed the difference in OS between the two groups in training and testing sets using Kaplan–Meier (KM) plots. The area under the curve (AUC) of the ROC curves was calculated to evaluate the predictive accuracy of the risk score (Blanche et al., 2013).

Univariate and multivariate Cox regression analyses were used to identify factors (risk score, clinicopathological stage, sex, age, and TMB) correlated with prognosis. Factors with corresponding hazard ratios and *p*-values are shown in the forest plot. A nomogram was established with independent prognostic factors by multivariate Cox regression analysis (*p* < 0.05).

### Pathway and functional enrichment analyses

To explore the functional and pathway differences between the low-risk and high-risk groups, DEGs were identified using the DESeq2 package (Love et al., 2014) (|log2FC| > 1 and padj < 0.05) and Gene Ontology (GO) and Kyoto Encyclopedia of Genes and Genomes (KEGG) enrichment analyses of the DEGs. GSEA was applied to analyze pathway enrichment analysis using the c2.cp.kegg gene set from MSigDB (Liberzon et al., 2015). Enrichment was performed using the clusterProfiler R package. Additionally, gene set variation analysis (GSVA) was used to estimate other pathway differences between the two groups, based on the c2.cp.kegg and hallmark gene sets from MSigDB (Yu et al., 2012).

### Drug sensitivity analysis

To investigate the association between the risk score and sensitivity to chemotherapeutics, we estimated the half-maximal inhibitory concentration (IC50) of anti-tumor agents using the pRRophetic package (Geeleher et al., 2014). Transcriptome and chemotherapy response data from the CellMiner database were downloaded and used to identify the correlations between drug sensitivity and expression levels of key m5C/m6A-related genes (Zheng et al., 2021).

### Statistical analyses

The statistical significance of the continuous variables in the two groups was estimated by the Wilcoxon rank-sum test. The Kruskal–Wallis test was used to estimate the statistical significance of continuous variables in two or more groups. The Fisher test was used to analyze the differences between the two groups of classified variables. KM curves were used to compare the differences in OS between the groups.

## Results

### Landscape of m5C/m6A-related genes in LUAD

**FIGURE 2:**
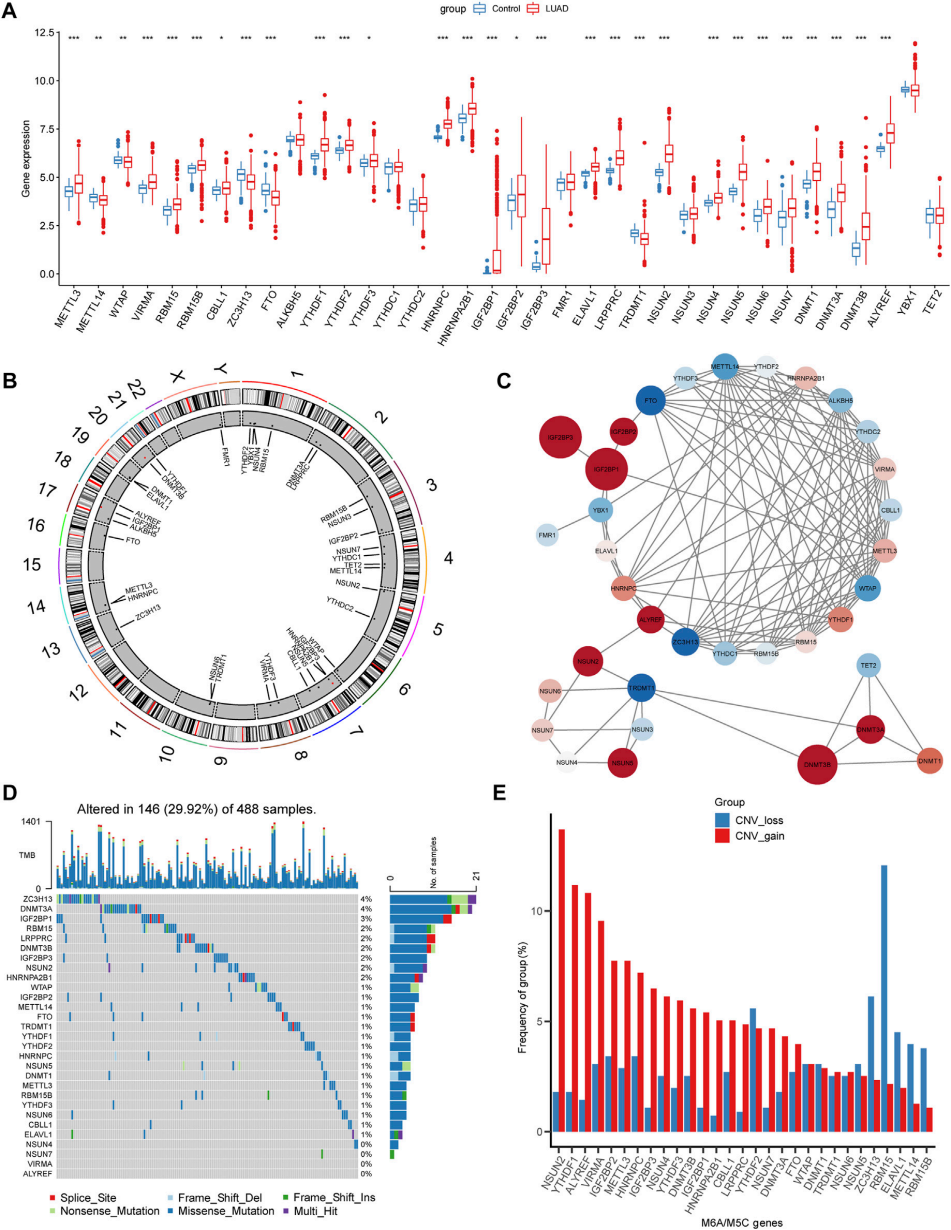
Landscapes of m5c/m6a-related genes in LUAD. **(A)** Boxplot illustrates the expression levels of 36 m5a/m6a-related genes in normal and LUAD tissues. **(B)** Chromosome locations of 36 m5a/m6a-related genes. **(C)** PPI network of m5a/m6a regulators. The color of nodes represents log2FC, and the size of nodes represents |log2FC| (log2FC was obtained as the result of difference analysis between TCGA normal samples and LUAD samples by DEseq2.) Red: log2FC >0; blue: log2FC < 0. **(D)** Waterfall diagrams show the mutation frequency of m5a/m6a-related genes. **(E)** CNV frequency of m5a/m6a-related genes. Red bars: CNV gain; blue bars: CNV loss. (*: *p* < 0.05, **: *p* < 0.01, and ***: *p* < 0.001).

First, we determined the expression levels of 36 m5C/m6A regulators in normal LUAD samples and the boxplot, and 29 regulators showed significant differences (Figure 2A).

The chromosomal locations of m5C/m6A regulators are shown in Figure 2B. The PPI network plotted the m5C/m6A regulators with an interaction score of 0.7 (Figure 2C). Red nodes, such as IGF2BP1, IGF2BP3, and DNMT3B, were notably upregulated in LUAD. Blue nodes, such as FTO, TRDMT1, and ZC3H13, indicate genes notably downregulated in LUAD. In TCGA-LUAD cohort, 146 samples (29.92%) had m5C/m6A-related gene alterations (Figure 2D). ZC3H13 and DNMT3A showed the highest alteration frequencies (4%). HNRNPA2B1, NSUN2, IGF2BP3, RBM15, LRPPRC, DNMT3B, and IGF2BP1 showed alteration frequencies ranging from 2% to 3%. The gene alteration frequency of the other genes ranged from 0% to 1%. CNV gain and loss of differentially expressed regulators are shown in Figure 2E. NSUN2, YTHDF1, ALYREF, and VIRMA showed prominent alterations in CNV gain.

### Characterization of m5C/m6A subtypes

**FIGURE 3:**
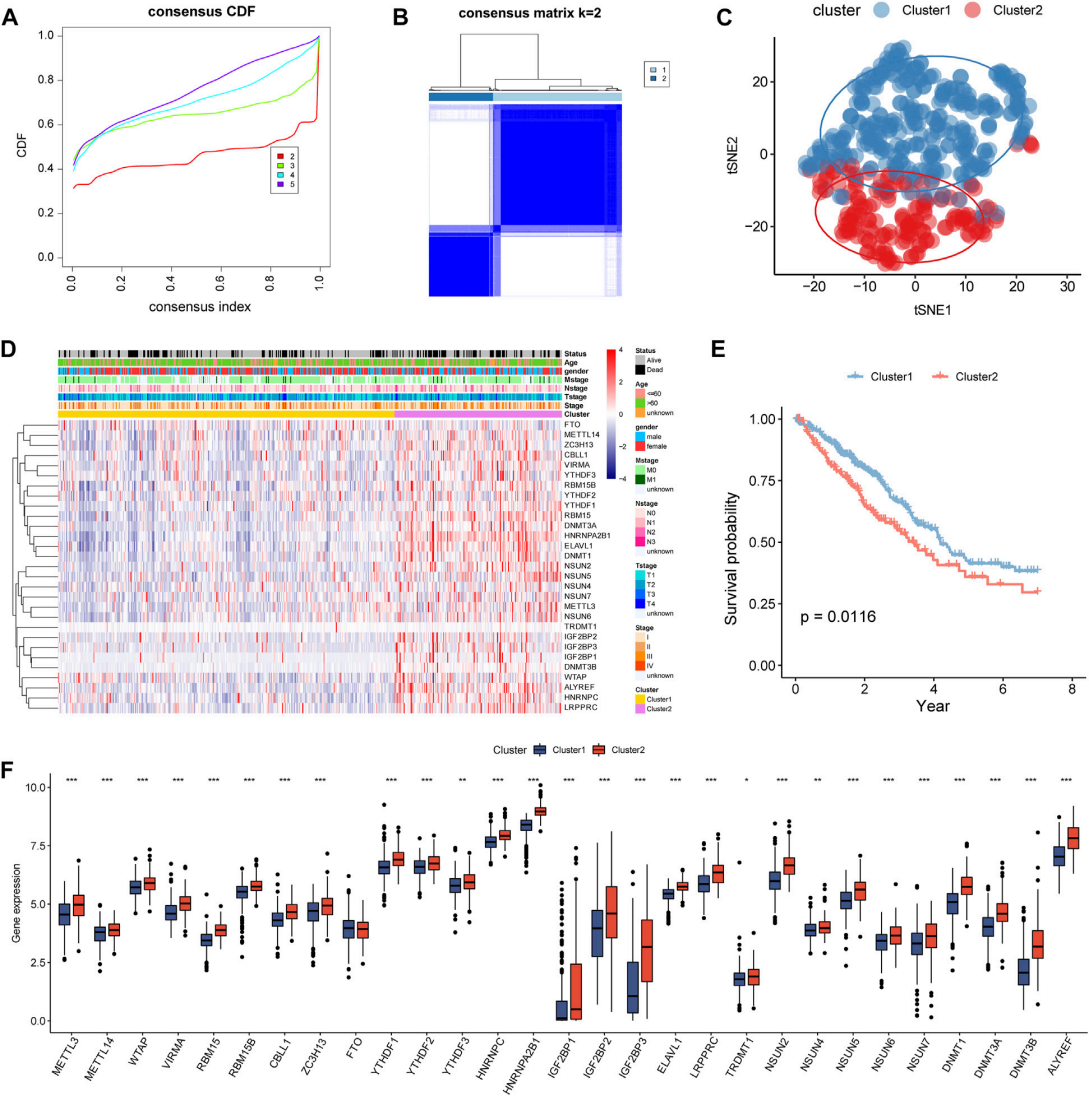
Identification of m5c/m6a modification subtypes in LUAD. **(A)** CDF for k = 2–5. **(B)** Heatmap of TCGA LUAD when k = 2. **(C)** *t*-SNE analysis for consensus clustering. **(D)** Expression of m5c/m6a-related genes in distinct m5c/m6a subtypes. **(E)** Survival analysis of LUAD patients for two subtypes. **(F)** Boxplots illustrating the expression differences of m5c/m6a-related genes between the two m5c/m6a subtypes.

According to the 29 differentially expressed m5C/m6A regulators, LUAD samples were divided into clusters (*k* = 2–5) by consensus cluster analysis (Figure 3A,B). Two subtypes were identified: 332 samples in cluster1 and 165 samples in cluster2. In the t-SNE plot, LUAD samples from cluster1 and cluster2 were distinguished by the expression of m5C/m6A-related genes (Figure 3C). The two clusters revealed marked differences in the expression levels of m5C/m6A-related genes, with cluster2 having a higher overall expression than cluster1 (Figure 3D). In addition, age and survival status were significantly different between the two clusters (*p* < 0.05). As shown in Figure 3E, cluster1 patients had a longer survival time than cluster2 patients (Figure 3E). Among the 29 m5C/m6A-related genes, 28 regulators showed significant differences between cluster1 and cluster2, which indicated that distinct m5C/m6A modification subtypes existed in LUAD.

**FIGURE 4:**
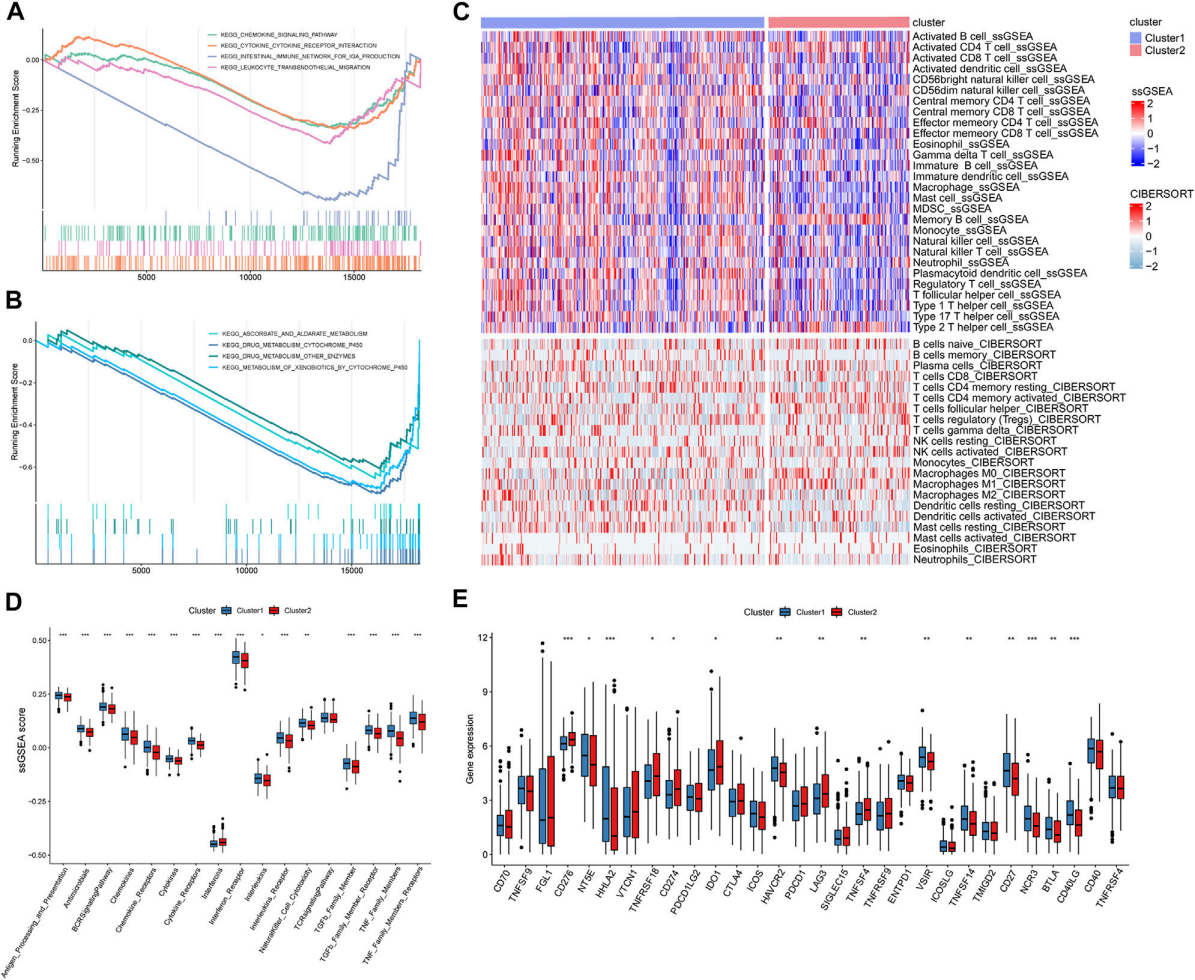
GSEA and immune landscape of distinct m5c/m6a modification subtypes. **(A,B)** GSEA of pathways related to different subtypes based on the C2 gene set. **(C)** Immune cell landscape of two m5c/m6a subtypes. **(D)** Boxplots comparing the ssGSEA scores of different molecular subtypes. **(E)** Boxplots comparing immune checkpoint gene expression of different molecular subtypes. (*: *p* < 0.05, **: *p* < 0.01, and ***: *p* < 0.001).

To identify differences in function and pathways between m5C/m6A subtypes, we performed GSEA. Metabolism-related pathways, such as drug metabolism–cytochrome p450, drug metabolism–other enzymes, and ascorbate and aldarate metabolism, were enriched in cluster1 (Figure 4A). Immune-related pathways such as the cytokine–cytokine receptor interaction, leukocyte transendothelial migration, chemokine signaling pathway, and intestinal immune network for IGA production were significantly enriched in cluster1 (Figure 4B). We used two algorithms to analyze the association between immune cells and m5C/m6A modification subtypes and visualized the results using heat maps (Figure 4C). In general, cluster1 had higher levels of infiltrating immunocytes than cluster2. As for the ssGSEA score of immune functions (Figure 4D), antigen processing and presentation, antimicrobials, the BCR signaling pathway, chemokines, chemokine receptors, cytokines, cytokine receptors, interferon receptor, interleukins, interleukin receptors, natural killer cell cytotoxicity, TGFb family member, TGFb family member receptors, TNF family members, and TNF family members were enhanced in cluster1. Interferon scores were higher in cluster2 than those in cluster1. Additionally, we noticed that 15 immune checkpoints were significantly different between different m5C/m6A subtypes (Figure 4E). CD276, TNFRSF18, CD274 (PD-L1), IDO1, LAG3, and TNFSF4 were highly expressed in cluster2. NT5E, HHLA2, HAVCR2, VSIR, CD27, NCR3, BTLA, CD40LG, and TNFSF14 were highly expressed in cluster1.

### Construction and evaluation of the m5C/m6A regulator-related prognostic model

**FIGURE 5:**
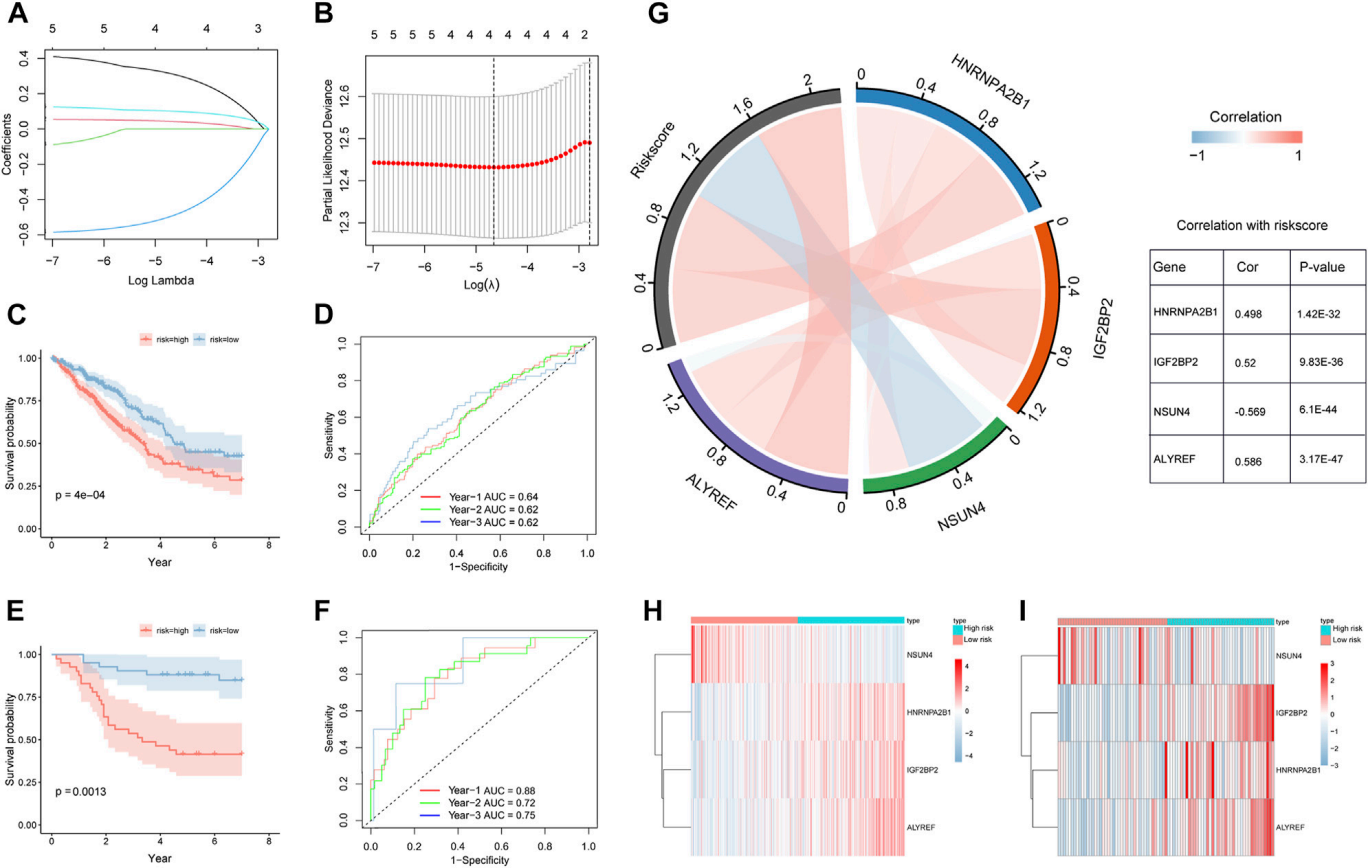
Construction and validation of m5C/m6A regulator-related prognostic model. **(A,B)** LASSO-Cox regression model by 10-fold cross-validation for OS in LUAD. **(C)** KM curves for the OS of high-risk and low-risk groups in TCGA training set. **(D)** Time ROC for OS in TCGA training set. **(E)** KM curves for the OS of high- and low-risk groups in the GEO testing set. **(F)** Time ROC for OS in the GEO training set. **(G)** Correlation chord plot of HNRNPA2B1, IGF2BP2, NSUN4, ALYREF, and risk score. **(H,I)** Heatmaps of expression levels of HNRNPA2B1, IGF2BP2, NSUN4, and ALYREF in the high-risk and low-risk groups of the training and testing sets.

We used univariate Cox regression analysis to screen out prognostic m5C/m6A regulators based on TCGA-LUAD and GSE30219 datasets (*p* < 0.1) (Supplementary Table S4). The genes (HNRNPA2B1, IGF2BP2, ELAVL1, NSUN4, and ALYREF) were analyzed using LASSO-Cox regression (Figure 5A,B). We then performed a multivariate Cox regression analysis and obtained four prognostic m5C/m6A-related genes (Table 2). The m5C/m6a-related risk score = exp-HNRNPA2B1*0.383+ exp-IGF2BP2* 0.0548- exp-NSUN4*0.595+ exp-ALYREF*0.113. We divided the LUAD samples into high- and low-risk groups using the median risk score. In both the training and testing sets, the high-risk groups had a significantly lower OS than the low-risk groups (*p* < 0.05) (Figure 5C,E). The 1-year, 2-year, and 3-year AUCs in the training set were 0.64, 0.62, and 0.62, respectively (Figure 5D). The 1-year, 2-year, and 3-year AUCs in the testing set were 0.88, 0.72, and 0.75, respectively (Figure 5F). The correlation chord plot illustrates the correlation between HNRNPA2B1, IGF2BP2, NSUN4, and ALYREF and the risk score. The correlation coefficients between HNRNPA2B1, IGF2BP2, NSUN4, and ALYREF and the risk score were 0.498, 0.52, −0.569, and 0.586, respectively (*p* < 0.05). In addition, the four genes were not remarkably correlated (|cor| < 0.4) (Figure 5G). The expression levels of HNRNPA2B1, IGF2BP2, NSUN4, and ALYREF in the training and testing sets are illustrated in the heatmaps (Figure 5H,I). The expression of the four m5C/m6A genes in LUAD and normal tissues is shown in the Human Protein Atlas (HPA) database (Supplementary Figure S1).

**TABLE 2:** Multivariate Cox regression analysis.

Gene	HR	HR.95L	HR.95H	*p-*value
*HNRNPA2B1*	1.47	1.02	2.10	0.037
*IGF2BP2*	1.06	0.94	1.19	0.36
*NSUN4*	0.55	0.36	0.84	0.0053
*ALYREF*	1.12	0.87	1.44	0.38

### Pathway, function, and immune-related analyses of the risk score

**FIGURE 6:**
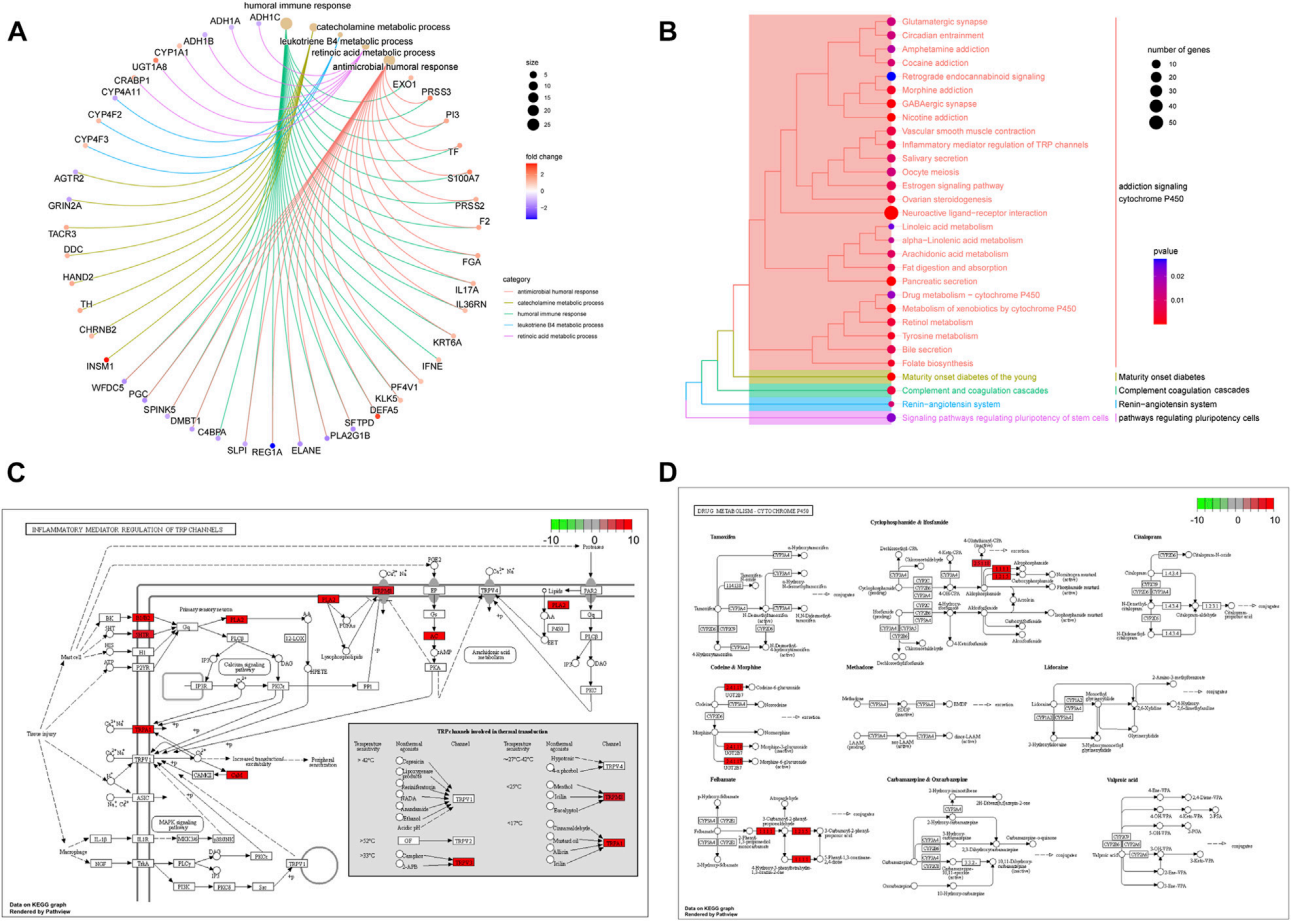
Functions and pathways associated with the risk score. **(A)** GO enrichment analysis of DEGs between high-risk and low-risk groups. **(B)** KEGG enrichment analysis of DEGs between high-risk and low-risk groups. **(C,D)** Signal pathway plots of hsa00982 and hsa00980.

To further explore the differences in functions and pathways between the high- and low-risk groups, KEGG and GO enrichment analyses were performed based on 882 DEGs (Supplementary Tables S5, S6). GO terms related to immunity and metabolism were significantly enriched, including the humoral immune response, catecholamine metabolic process, leukotriene B4 metabolic process, retinoic acid metabolic process, and antimicrobial humoral response (*p* < 0.05) (Figure 6A). KEGG pathways, such as addiction signaling cytochrome p450, maturity-onset diabetes, complement coagulation cascades, renin-angiotensin system, and pathways regulating pluripotency cells were enriched (*p* < 0.05) (Figure 6B). The enriched pathways (hsa00982 and hsa00980) are shown in pathway plots, and the related genes are labeled (Figure 6C,D).

**FIGURE 7:**
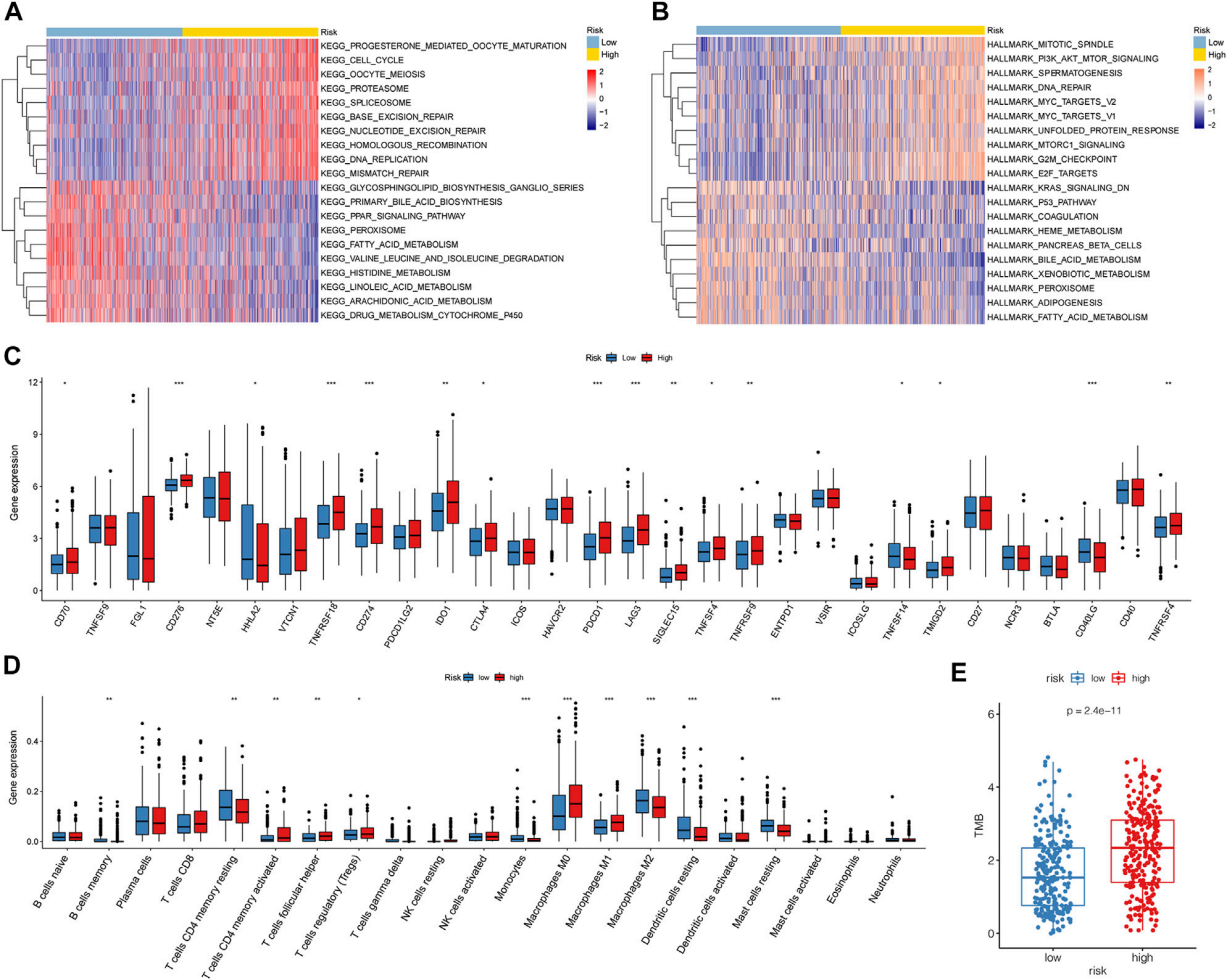
Biological characteristics, pathways, and immune-related analyses between high- and low-risk groups. **(A,B)** Heatmaps of GSVA between the different risk groups with C2 and hallmark gene sets. **(C)** Boxplots illustrating expression of immune checkpoints of the high- and low-risk groups. **(D)** Boxplots illustrating abundance of immune cells in the high- and low-risk groups. Red boxes: high-risk group; blue boxes: low-risk group. (*: *p* < 0.05, **: *p* < 0.01, and ***: *p* < 0.001). **(E)** High-risk group had higher TMB than the low-risk group (*p* = 1e-11).

To further investigate the pathways related to the risk score, GSVA was performed using C2 and hallmark gene sets. Compared to that in the low-risk group, the cell cycle, oocyte meiosis, DNA replication, and nucleotide excision repair were significantly enriched in the high-risk group. Metabolism-related pathways, such as drug metabolism, cytochrome p450, and fatty acid metabolism, were enriched in the low-risk group (Figure 7A). Among the hallmark gene sets, those related to the cell cycle, such as PI3K-AKT mTor, E2F, mitotic spindle, and DNA repair, were enriched in the high-risk group (Figure 7B). The P53 pathway, KRAS signaling (down), and fatty acid metabolism were enriched in the low-risk group. In addition, 16 immune checkpoint genes showed significant differences between the high- and low-risk groups. Among these were 13 immune checkpoints, namely, CD70, CD276, TNFRSF18, CD274, IDO1, CTLA4, PDCD1, LAG3, SIGLEC15, TNFSF4, TNFRSF9, TMIGD2, and TNFRSF4, which were highly expressed in the high-risk group (Figure 7C). The high-risk group had higher immune infiltration levels of CD4 memory-activated T cells, follicular helper T cells, Tregs, M0 macrophages, and M1 macrophages. The low-risk group showed higher immune infiltration levels of memory B cells, resting memory CD4 T cells, monocytes, M2 macrophages, resting dendritic cells, and resting mast cells (Figure 7D).

### Mutation landscapes of low- and high-risk groups

The GSVA results showed that DNA repair and mismatch repair pathways significantly differed between the low- and high-risk groups. DNA repair and mismatch repair pathways critically impact gene mutations in tumors. Therefore, we further compared the mutation profiles of samples in the low- and high-risk groups.

**FIGURE 8:**
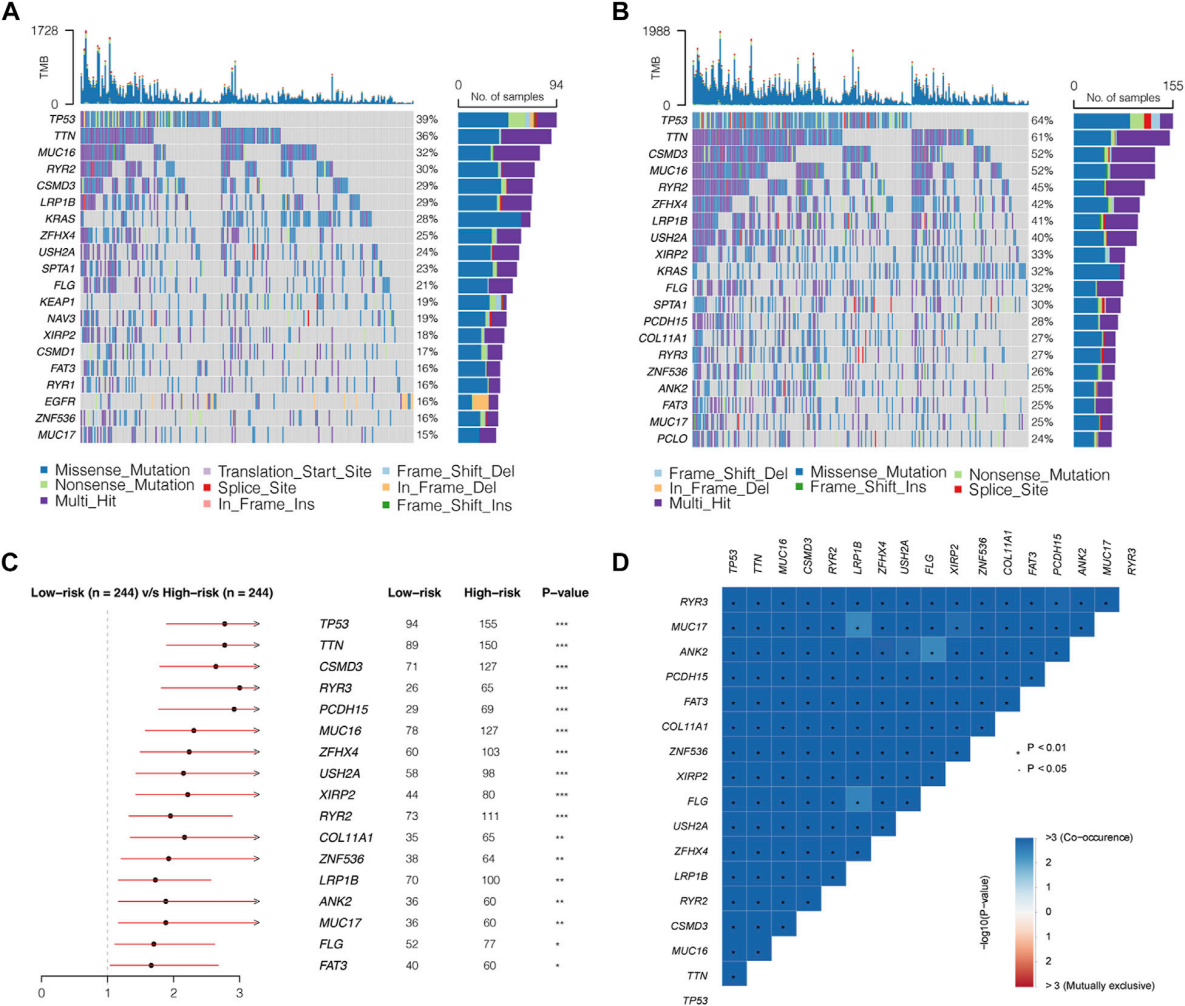
Mutation landscapes of low- and high- risk groups. **(A,B)** Waterfall diagrams displaying 20 genes with the highest mutation rates in the low- and high-risk groups. **(C)** Seventeen genes mutating differently in the two groups. **(D)** Correlations of 17 differently mutating genes. Red represents co-occurrence, and blue represents mutually exclusive.

First, we calculated the TMB of the two groups and found that the high-risk group had a higher TMB than the low-risk group (Figure 7E). A total of 20 genes with the highest mutation rates in the two groups were plotted using waterfall diagrams (Figure 8A,B). In general, the mutation rates of the 20 genes were higher in the high-risk group than in the low-risk group. In the forest plot, 17 genes with higher mutation rates in the high-risk group were *TP53*, *TTN*, *CSMD3*, *RYR3*, *PCDH15*, *MUC16*, *ZFHX4*, *USH2A*, *XIRP2*, *RYR2*, *COL11A1*, *ZNF536*, *LRP1B*, *ANK2*, *MUC17*, *FLG*, and *FAT3* (*p* < 0.05) (Figure 8C). Moreover, apparent co-occurrences were observed in 17 genes (Figure 8D).

### Clinical prediction model based on the m5C/m6A-related risk score

**FIGURE 9:**
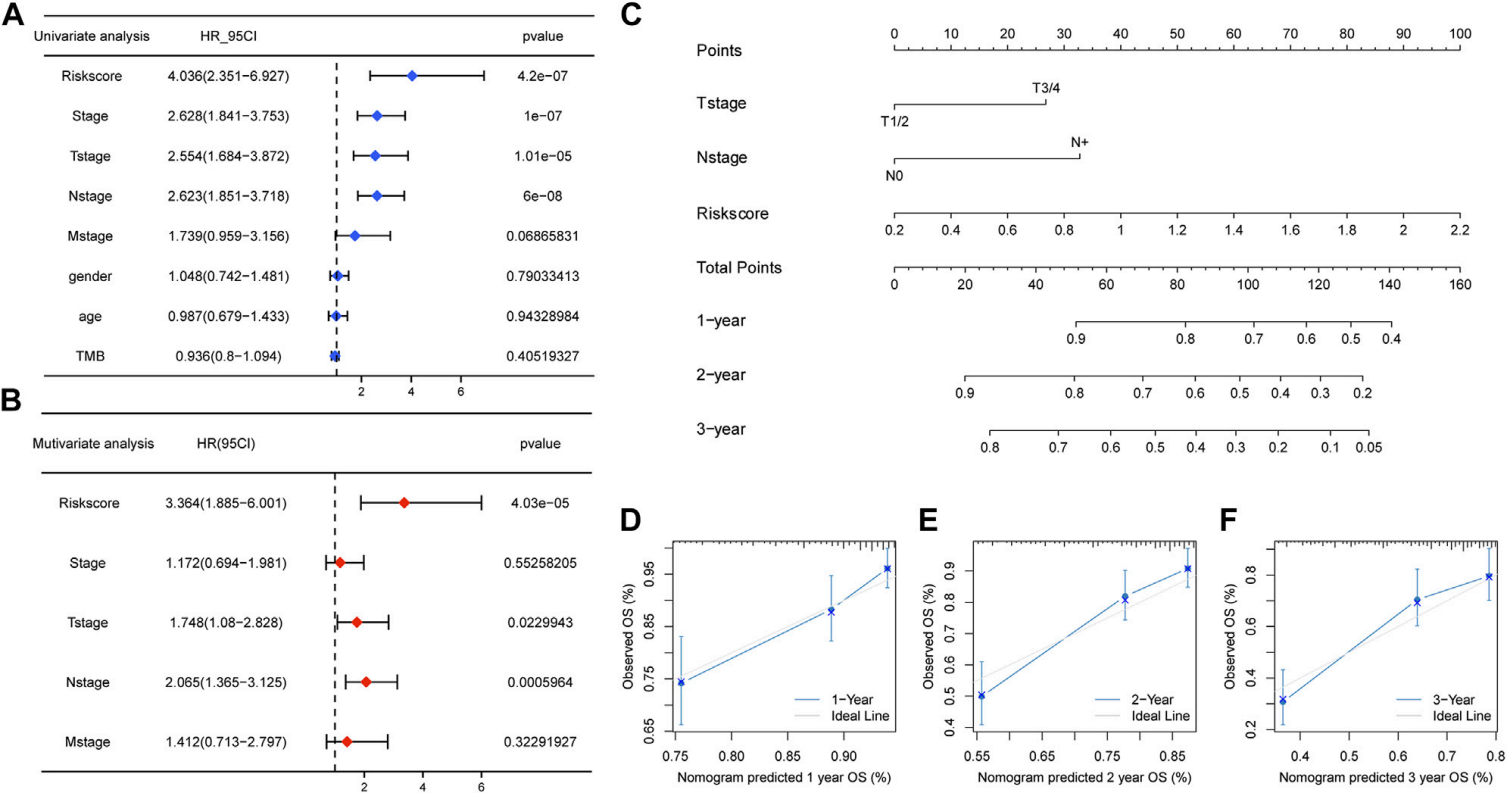
Clinical predicting model based on the risk score. **(A,B)** Univariate and multivariate Cox regression analyses for the risk score and clinical factors. The risk score, T stage, and N stage were independent prognostic factors for TCGA-LUAD patients. **(C)** Nomogram predicting for 1-, 2-, and 3-year OS with the independent prognostic factors. **(D–F)** 1 year, 2 years, and 3 years of calibration plots for the clinical predictive model.

Considering the predictive ability of the m5C/m6A-related risk score for LUAD prognosis, we attempted to identify whether the risk score could be an independent prognostic factor together with the pathological stage, T stage, N stage, M stage, age, sex, and TMB. The results of univariate Cox regression analysis illustrated the relationships between the factors and prognosis, and factors with *p* < 0.1 were incorporated into multivariate Cox regression analysis. The risk score, T stage, and N stage were independent prognostic factors for patients with LUAD (Figure 9A,B). A nomogram was constructed with independent prognostic factors for predicting 1-, 2-, and 3-year OS (Figure 9C). The C-index of the model was 0.72. Figure 9D–F showed the 1-, 2-, and 3-year calibration curves, respectively, which fitted well with ideal curves.

### Predictive potential of a risk score for immunotherapy response and chemotherapy sensitivity

**FIGURE 10:**
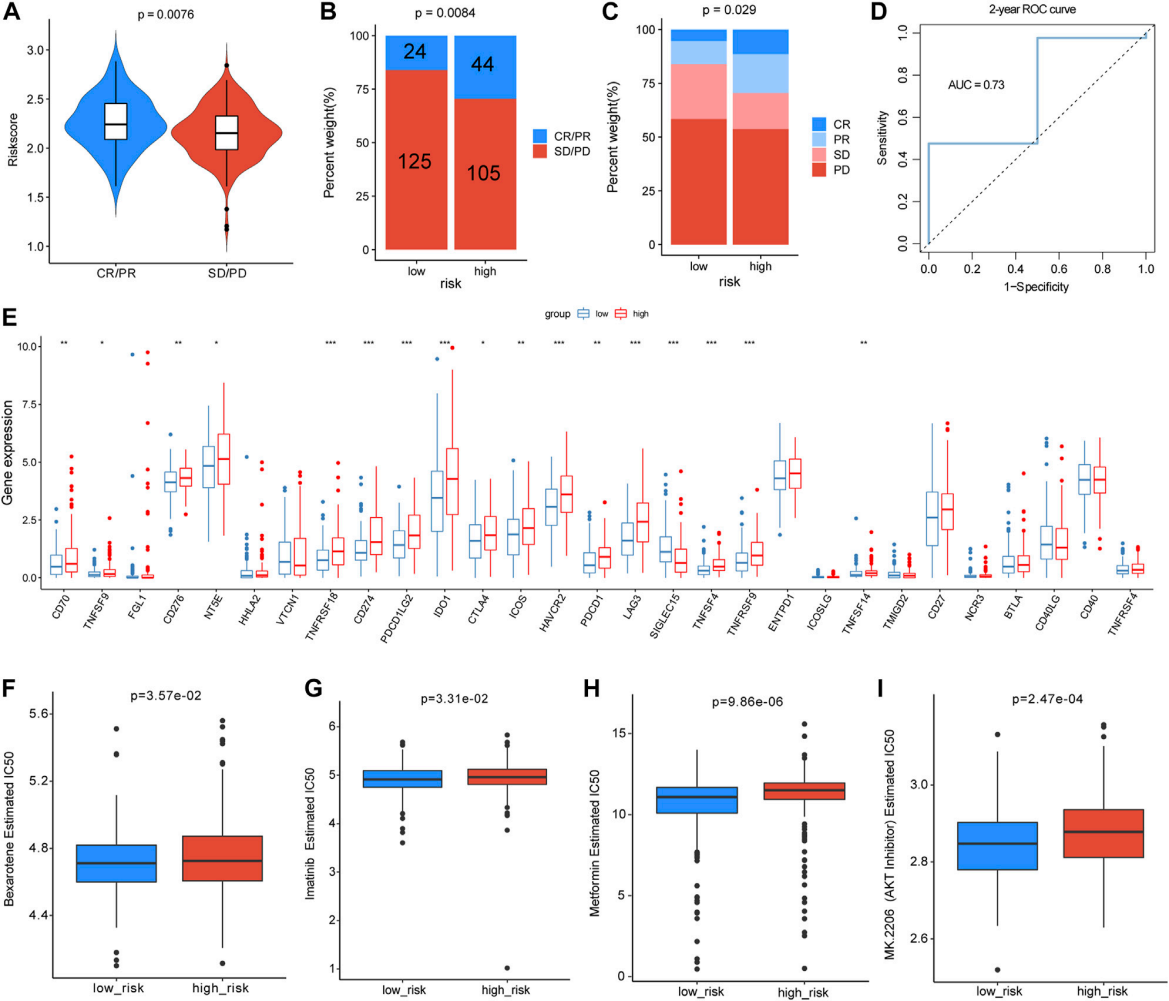
Analyses of risk scores for predicting immunotherapy efficacy and drug sensitivity. **(A)** Distribution of the risk score between patients with PD/SD and patients with PR/CR in the IMvigor210 dataset. **(B,C)** Proportion of patients with different immunotherapy responses in different risk groups. **(D)** Two-year ROC curve for estimating the predictive efficacy of the risk score. **(E)** Differences in expression levels of immune checkpoints between different risk groups. **(F–I)** Drug sensitivity differences of bexarotene, imatinib, metformin, and AKT inhibitor in the two groups.

As the m5C/m6A-related risk score was closely correlated with immune-related functions and checkpoints, we further investigated its relationship with the immunotherapy response. In the IMvigor210 dataset, patients with progressive disease (PD) or stable disease (SD) had lower risk scores than those with partial response (PR) and complete response (CR) (*p* = 0.0076) (Figure 10A). According to the median risk score, 298 patients from IMvigor210 with complete clinical data were divided into low- and high-risk groups. The proportion of SD or PD was higher in the low-risk group than in the high-risk group (*p* = 0.0084 and *p* = 0.029, respectively) (Figure 10B,C). To estimate the predictive efficacy of the risk score for immunotherapy response, we performed a 2-year ROC curve, and the AUC was 0.73 (Figure 10D). In total, the expression of 17 immune checkpoint genes differed between the low-risk and high-risk groups, and 16 immune checkpoints were higher in the high-risk group (Figure 10E). In the analyses of drug sensitivity between the two groups, patients in the high-risk group had lower sensitivity to bexarotene (*p* = 3.57e-02), imatinib (*p* = 3.31e-02), metformin (*p* = 9.86e-06), and AKT inhibitors (*p* = 2.47e-04) (Figure 10F–I). Additionally, we also analyzed the correlations between the m5C/m6A-related genes and the sensitivity of anti-tumor drugs using the CellMiner database and identified the 20 most correlated drugs (|Cor| > 0.4) (Supplementary Figure S2). ALYREF was positively correlated with the sensitivity of 5−fluorodeoxyuridine, floxuridine, irinotecan, nelarabine, triethylenemelamine, thiotepa, and LMP−400. IGF2BP2 was negatively correlated with sensitivity to dexrazoxane, SR16157, etoposide, teniposide, raloxifene, XK−469, idarubicin, bendamustine, and fulvestrant. NSUN4 expression was negatively correlated with sensitivity to vorinostat. HNRNPA2B1 was positively correlated with the sensitivity to ifosfamide, nelarabine, and chelerythrine.

## Discussion

As a crucial epigenetic modification, methylation plays a role in the modulation of gene expression and affects various diseases. Previous studies have found that RNA modification of m5C and m6A can affect malignant biological processes by regulating the proliferation and migration of tumor cells. In a previous study, different lung cancer subtypes clustered by the signature of 13 m6A regulators showed significant differences in prognosis and pathological stage (Liu et al., 2020b). In gastric cancer, decreased methylation levels can augment the PI3K-AKT pathway and promote the invasion and proliferation of gastric cells. ALKBH5 acts as a pivotal m6A eraser and promotes the proliferation and invasion of LUAD cells (Chao et al., 2020). m5c-related genes are involved in eukaryotic growth and evolution, and m5c regulators are associated with the tumor microenvironment and prognosis of patients with LUAD (Chen et al., 2021b). The m5C writer NSUN2 was demonstrated to enhance the progression of squamous cell carcinoma by stabilizing LIN28B-dependent GRB2, which could activate the AKT and RTK signaling pathways (Su et al., 2021). However, the cellular and biological mechanisms of m5C/m6A-related genes in developing and treating LUAD remain unclear. Based on the results of previous studies, we sought to construct a robust risk model to guide clinical decision-making for patients with LUAD.

We collected 36 m5C/m6A-related genes from previous studies in this study. The expression levels and mutation profiles of 36 m5C/m6A-related genes were analyzed, and 29 differentially expressed m5C/m6A regulators were identified. Based on the differentially expressed m5C/m6A-related genes, we identified distinct m5C/m6A-related modification subtypes in LUAD by unsupervised clustering and compared the differences in functions and pathways between different clusters. Using univariate Cox regression, LASSO regression, and multivariate Cox regression, prognostic m5C/m6A regulators were identified, and a prognostic risk model was constructed and validated using GSE30219. We found that cluster1 had lower m5C/m6A regulator expression, higher OS, higher immune activity, and an abundance of infiltrating immune cells than cluster2. Using multiple enrichment analysis methods, the risk score was demonstrated to be closely correlated with immune and metabolic pathways. High-risk LUAD patients had a worse prognosis, higher immune checkpoint expression, and higher TMB than low-risk LUAD patients. The predictive value of the risk score for immunotherapy response and drug sensitivity was also illustrated. We concluded that the m5C/m6A-related risk score could be a crucial marker for predicting prognosis and immunotherapeutic response, which might improve personalized treatment for patients with LUAD.

The complexity of immune cell infiltration in the tumor microenvironment is one of the leading causes of LUAD treatment efficacy. According to bioinformatics analysis studies, m6A regulators are correlated with tumor prognosis and the immune microenvironment (Fan et al., 2022; Xiong et al., 2022). Two m5C modification patterns had different immune cell-infiltrating subtypes, illustrating that m5C might regulate the LUAD immune microenvironment (Chen et al., 2021b). Intratumor immune cells play a crucial role in the TME and affect the prognosis and pathogenesis of LUAD (Jiang et al., 2020).

Therefore, it is crucial to explore the linkage between m5C/m6A regulators, infiltration of immune cells, and treatment of LUAD. We found that 29 m5C/m6A regulators (METTL3, METTL14, WTAP, VIRMA, RBM15, RBM15B, CBLL1, ZC3H13, FTO, YTHDF1, YTHDF2, YTHDF3, HNRNPC, HNRNPA2B1, IGF2BP1, IGF2BP2, IGF2BP3, ELAVL1, LRPPRC, TRDMT1, NSUN2, NSUN4, NSUN5, NSUN6, NSUN7, DNMT1, DNMT3A, DNMT3B, and ALYREF) were significantly different between the LUAD and normal samples. The two m5C/m6A subtypes identified based on these genes by unsupervised clustering were used to define the immune activity status of LUAD. Cluster1, with a lower expression of m5C/m6A regulators, had higher immune function scores and degrees of infiltrating immune cells than cluster2. Immune cell infiltration plays a vital role in immunotherapy. Patients with different immune infiltration conditions show differing clinical and immunotherapeutic benefits (Zeng et al., 2019; Lebid et al., 2020). The two m5C/m6A modification subtypes, accompanied by distinct immune phenotypes, improve our understanding of the immune microenvironment for the precise application of immunotherapy.

The risk score model was constructed with HNRNPA2B1, IGF2BP2, NSUN4, and ALYREF, and the high-risk group was closely related to the high expression of immune checkpoint genes and high TMB in TCGA-LUAD samples. In total, 13 checkpoint genes were upregulated in the high-risk group, including CTLA4, LAG3, (PD1) PDCD1, and (PDL1) CD274, as markers of T-cell exhaustion. In addition, the proportion of patients who benefited from the PD-L1 blockade was higher in the high-risk group than that in the low-risk group. Immune checkpoint genes resist elimination mediated by immunity, which is the main reason for the immune escape from lung cancer (Cheng et al., 2017). ICIs as novel anti-tumor drugs have shown promising therapeutic efficacy in some cancers, such as melanoma, non-small cell lung cancer, and urinary system cancers (Hamid et al., 2013; Sharma and Allison, 2015). In addition, studies have revealed that the efficiency of ICIs for NSCLC ranges from 15% to 20% (Wu et al., 2018). From a clinical perspective, increased expression of immune checkpoints and high TMB are significant indicators of immunotherapy. Patients with high TMB or expression levels of checkpoint genes benefit more from immunotherapeutic agents in multiple types of cancers (Cinausero et al., 2019). Mismatch repair deficiency is a reliable predictor of response to anti-PDL1 agents in patients with malignancies (Wang et al., 2018). These findings are consistent with those of the present study. As for differentially mutated genes between the low- and high-risk groups, 19 genes were highly mutated in the high-risk group. The mutation frequency of the tumor suppressor TP53 was significantly higher in the high-risk. Dong et al. (2017) indicated that mutations in TP53 and KRAS, especially co-occurring mutations, enhance the expression of immune checkpoints and are remarkable factors for ICIs in patients with LUAD. In summary, based on the differences in mutation profiles, immune checkpoints, and responses to immunotherapy between the low- and high-risk groups, we suggest that the risk score could be an effective tool for predicting immunotherapy efficacy.

We also found that the low-risk group had greater infiltration of M2 macrophages and fewer M1 macrophages. Macrophages have two major phenotypes: pro-inflammatory M1 and tumor-promoting M2 macrophages. However, a study by Mehrdad et al. showed that the infiltration of CD204 M2 macrophages improved the prognosis of patients with NSCLC (Rakaee et al., 2019). In another study on the immune microenvironment of LUAD, M2 macrophages were enriched in patients with prolonged survival and lower mutation load(Wu et al., 2021). These findings are consistent with our research, and the relationship between macrophage phenotypes and LUAD prognosis requires further exploration. Enrichment analyses revealed that mismatch repair, humoral immune response, drug metabolism–cytochrome p450, leukotriene B4 metabolic process, PI3K-AKT, mitotic spindle, and DNA repair were significantly different among the different risk groups. We inferred that prognostic m5C/m6A regulators, including HNRNPA2B1, IGF2BP2, NSUN4, and ALYREF, might be involved in immune-, metabolic-, and proliferation-related functions in LUAD. Among the prognostic RNA modification regulators, HNRNPA2B1 and NSUN4 were considered independent prognostic factors for LUAD. It has been reported that nuclear HNRNPA2B1 augmentation can induce an innate immune response by amplifying IFN-α/β production (Wang et al., 2019). In an experimental study, HNRNPA2B1, regulated by CACNA1G-AS1, increased the epithelial–mesenchymal transition of NSCLC cells (Yu et al., 2018). ALYREF may act as a poor prognosis biomarker in patients with bladder cancer and is involved in glycolysis and cell proliferation by regulating PKM2 (Wang et al., 2021b). In addition, NSUN4 is upregulated in hepatocellular cancer and shows excellent performance as a biomarker for the prognosis of hepatocellular carcinoma (Cui et al., 2021). Several cancer-promoting and inflammation-related functions have been proposed for IGF2BP2, including the promotion of tumor growth and induction of macrophage polarization (Wang et al., 2021c; Wang et al., 2021d). Further studies are warranted to investigate the immune- and metabolism-related functions of the four prognostic m5C/m6A regulators in LUAD.

In contrast to previous studies on the m5C signature or m6A signature in LUAD, we first investigated the integration of m5C and m6A regulators and assessed significant parameters for immunotherapy, including TMB, 30 common immune checkpoints, and immune cell infiltration. In the present study, high-risk patients had high TMB, increased expression of immune checkpoints, and high sensitivity to immunotherapy, suggesting that the risk score formula could predict immunotherapy in LUAD. Understanding the mechanisms of the metabolic processes and immune responses mediated by m5C/m6A regulators may enhance the therapeutic effects of LUAD. The results of our research provide an m5C/m6A risk model with excellent clinical significance. Additionally, high-risk patients were found to have an observably low OS and poor therapeutic sensitivity to imatinib and AKT inhibitors, indicating that it is necessary to pay more attention to disease progression and drug resistance in high-risk LUAD patients. Future research needs to focus more on the role of m5C and m6a in immunity and drug resistance in LUAD.

Our study has some limitations. First, more comprehensive clinical factors should be included to determine whether the risk score is an independent prognostic factor for LUAD. Second, LUAD cohorts receiving immunotherapy were required to verify the accuracy and stability of the risk score in our study. Third, the study lacked experimental validation. Therefore, we are collecting clinical samples of LUAD, and further *in vitro* and *in vivo* experiments may be used to investigate the biological functions of prognostic m5C/m6A-related genes.

In summary, we identified two m5C/m6A modification subtypes associated with different immune phenotypes and demonstrated the value of the m5C/m6A-related risk score for estimating prognosis, drug resistance, and immunotherapy efficacy. The proposed m5C/m6A-related risk score model may assist in prognosis evaluation and improve treatment efficacy for patients with LUAD.

## Data Availability

The original contributions presented in the study are included in the article/Supplementary Material; further inquiries can be directed to the corresponding author.
