# Supplementary figures and images for: Identification of a novel m5C/m6A-related gene signature for predicting prognosis and immunotherapy efficacy in lung adenocarcinoma
Source: Front Genet. 2022 Sep 30;13:990623. doi: 10.3389/fgene.2022.990623 (PMC9561349; doi:10.3389/fgene.2022.990623)

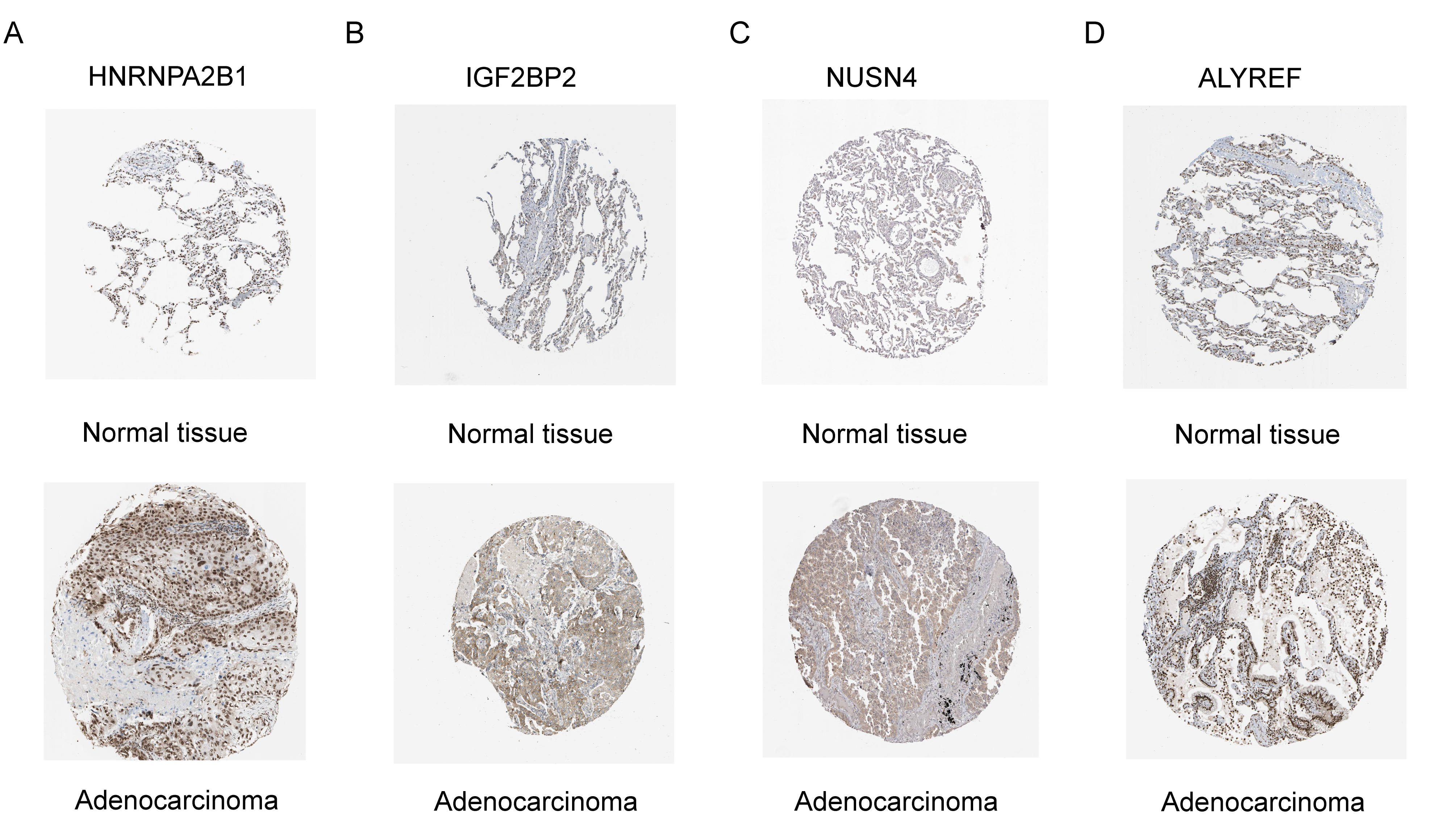

Supplement: Supplementary file 1 [file Image2.TIF]

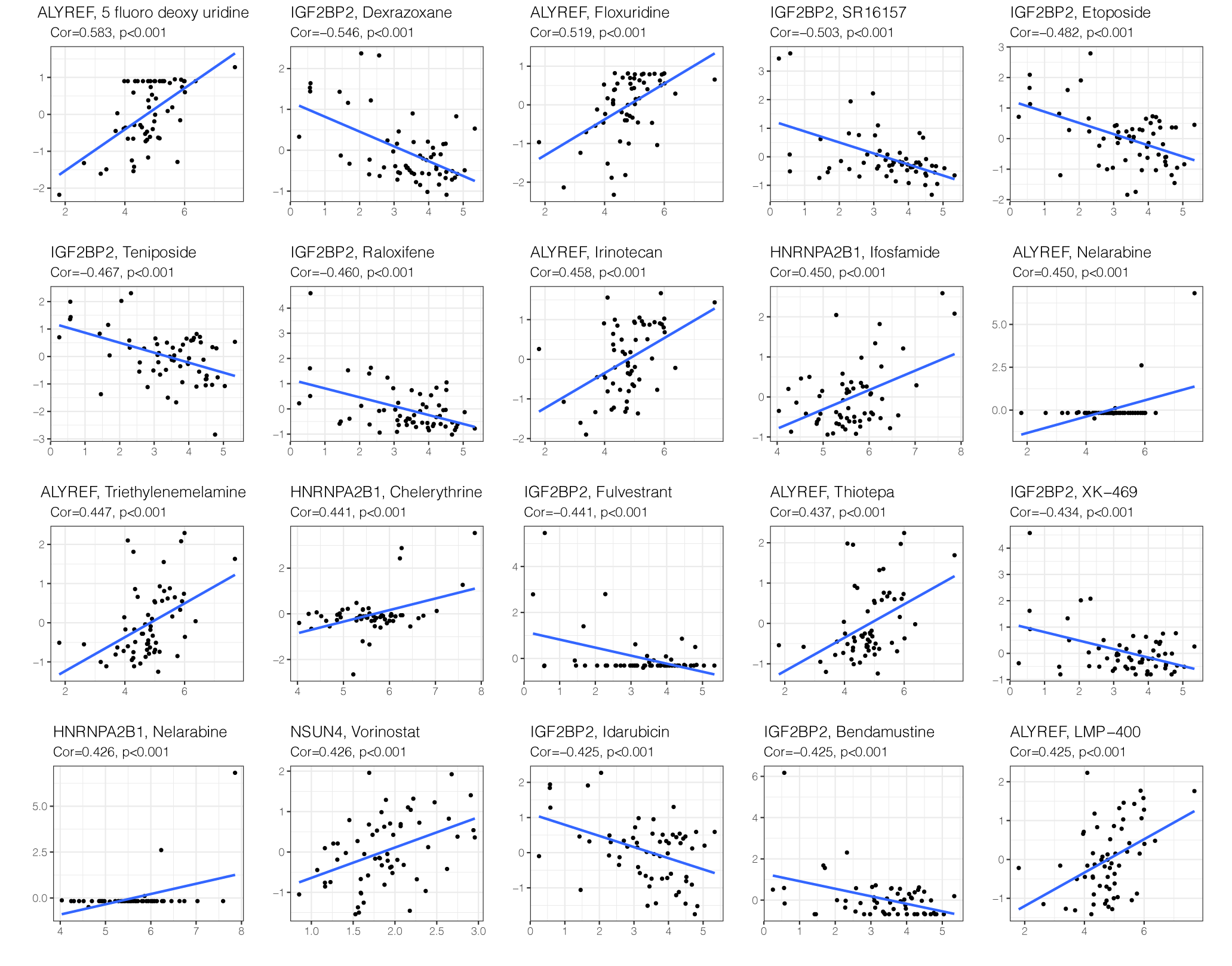

Supplement: Supplementary file 2 [file Image1.TIF]
