# Supplementary material for: Identification of a novel m5C/m6A-related gene signature for predicting prognosis and immunotherapy efficacy in lung adenocarcinoma
Source: Front Genet. 2022 Sep 30;13:990623. doi: 10.3389/fgene.2022.990623 (PMC9561349; doi:10.3389/fgene.2022.990623)
Supplement: Supplementary file 3 [file DataSheet2.docx]

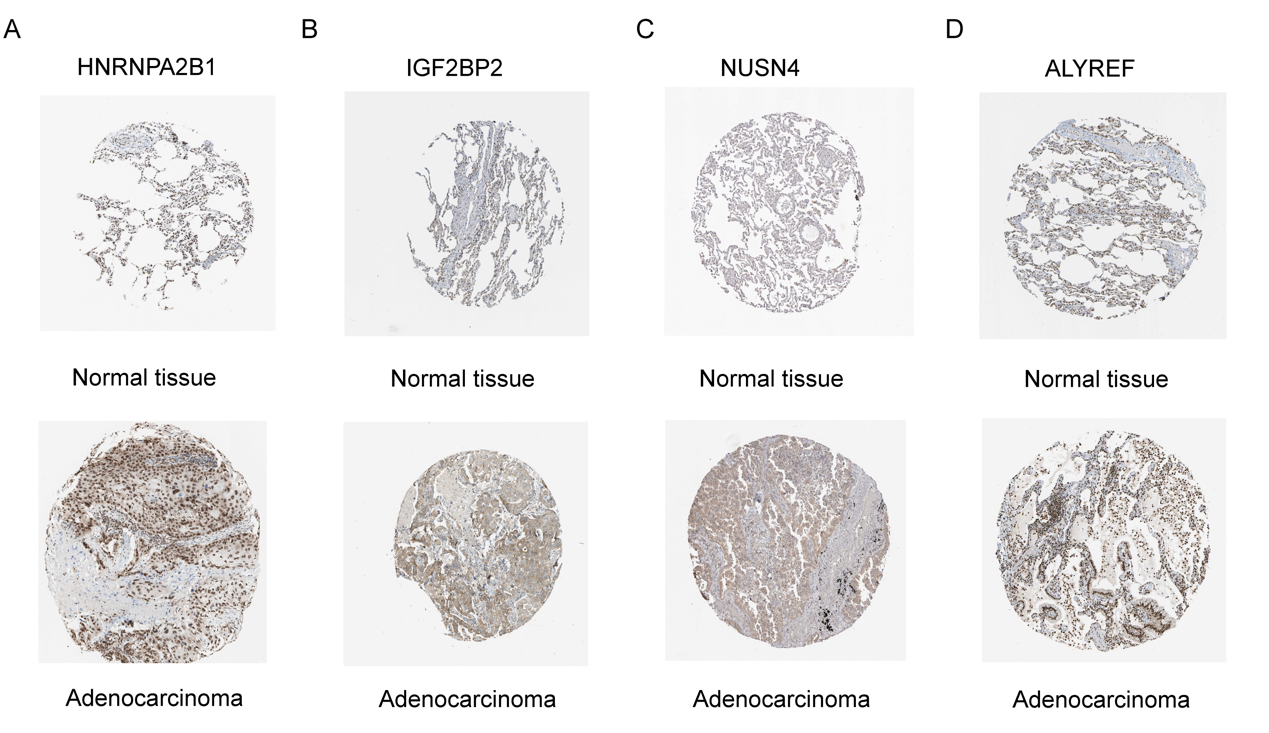


**Supplementary Figure 1** | Expression of the four prognostic m5C/m6A genes in LUAD and normal tissues. **(A-D)** Immunohistochemical staining for HNRNPA2B1, IGF2BP2, NSUN4 and ALYREF from HPA database.


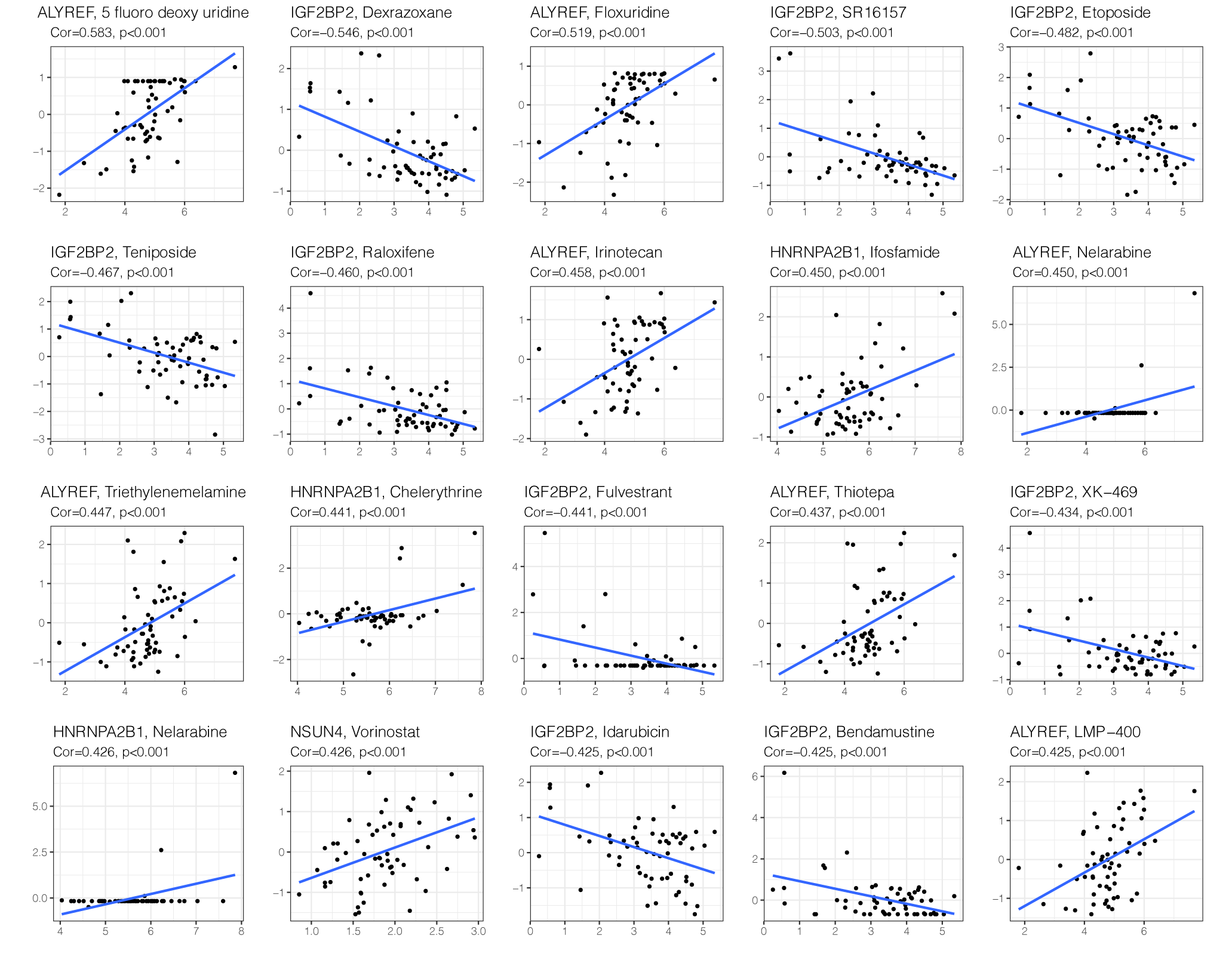


**Supplementary Figure 2** | Drug sensitivity analysis. Drug sensitivity analysis of prognostic m5C/m6A regulators (HNRNPA2B1, IGF2BP2, NSUN4 and ALYREF).
